# Insecticide resistance status in *Culex quinquefasciatus* in Benin

**DOI:** 10.1186/s13071-015-0638-3

**Published:** 2015-01-13

**Authors:** Agnes Yadouléton, Kefilath Badirou, Ramziath Agbanrin, Hanna Jöst, Roseline Attolou, Ramasamy Srinivasan, Gil Padonou, Martin Akogbéto

**Affiliations:** Centre de Recherche Entomologique de Cotonou (CREC), 06 BP 2604, Cotonou, République du Bénin; Ecole Normale Supérieure de Natitingou-Université de Parakou, Parakou, Benin; Université d’Abomey-Calavi, Faculté des Sciences et Techniques, Cotonou, Benin; Bernhard Nocht Institute for Tropical Medicine, WHO Collaborating Centre for Arbovirus and Haemmorrhagic Fever Reference and Research, Bernhard-Nocht-Strasse 74, Hamburg, Germany; German Centre for Infection Research, partner site Hamburg-Luebeck-Borstel, Hamburg, Germany; AVRDC-The World Vegetable Center, Shanhua, Tainan, 74151 Taiwan

**Keywords:** *Culex quinquefasciatus*, Insecticide, Resistance, Agriculture, Benin

## Abstract

**Background:**

*Culex quinquefasciatus*, an arboviral and filarial vector, is present year round in several cities of the Republic of Benin. There is more information on the resistance status to malaria vectors compared to Culicines. It is therefore unfortunate that the international focus is on Anopheles control and not so much done against *Cx. quinquefasciatus*, a rather more resilient mosquito to many insecticides that deserves attention. The present study aims to assess the resistance status of *Cx. quinquefasciatus* to carbamates, pyrethroids and organochlorine and discuss the implications for vector control in four contrasting localities of the country.

**Methods:**

Four contrasting localities of the country were selected for mosquito collection during the dry season based on their variation in agricultural production, use of insecticides and/or ecological settings. Bioassay were performed on adults collected from the field to assess the susceptibility of *Cx. quinquefasciatus* to insecticide-impregnated papers (permethrin 0.75%, delthamethrin 0.05%, DDT 4%, and bendiocarb 0.1%) following WHOPES guidelines. Molecular assays were carried out to detect the presence of *knock down resistance (kdr)* and *acetylcholinesterase* (*ace. 1)* mutations in surviving specimens using PCR techniques.

**Results:**

WHO diagnostic tests showed high frequency of resistance in *Cx. quinquefasciatus* to permethrin (ranging from 4 to 24% mortality), deltamethrin (24 to 48%), DDT (4 to 12%) and bendiocarb (60 to 76%) in the four selected areas. This was consistent with the presence of target site insensitivity due to *kdr and ace.1* mutations, which were significantly higher in areas where farmers used insecticides for pests control than in areas where no insecticides were used (p < 0.05.).

**Conclusion:**

These findings showed that wild populations of *Cx. quinquefasciatus* have developed resistance against pyrethroids, organochlorine and carbamate. This situation of resistance may seriously jeopardize the efficacy of Insecticide Residual Spray (IRS) and Long-Lasting Insecticide nets (*LLINs*) on which, most African countries including Benin, rely to reduce malaria transmission.

## Background

*Culex quinquefasciatus* is a major biting nuisance, particularly in urban areas where it thrives in wet pit latrines, blocked open drains, and polluted puddles [1]. In Benin, *Cx. quinquefasciatus* is a common mosquito that lives close to people due to the presence of large number of *Cx. quinquefasciatus* breeding sites [2].

*Cx. quinquefasciatus* is a member of the *Culex pipiens* complex Linnaeus and one of the main subspecies found in Africa [3,4]. *Cx. quinquefasciatus* (Diptera: Culicidae) is widely distributed in tropical and subtropical areas and is the most important vector of filarial parasite *Wuchereria bancrofti*, although *Anopheles gambiae* s.l and *An. funestus* also play a role in selected areas [5-9].

In Africa, the prevalence of lymphatic filariasis (LF) is especially striking, affecting over 40 million people in the sub-Saharan region [2]. The LF program established in 1994 with a mass drug administration (MDA) campaign to treat sick people in 2001 was associated with the launch of the Global Program to Eliminate LF (GPELF) in many African countries by the World Health Organization (WHO) and proved successful [10]. The free distribution of Insecticide-Treated Nets (ITN) and the implementation of Indoor Residual Spraying (IRS) as vector control methods against malaria have contributed to the great success of this program.

However, successful implementation of these vector control strategies requires sound knowledge of vector distributions, biology and changing trends on susceptibility status to available insecticide compounds.

Indeed, it is possible that these insecticides used to Eliminate LF (GPELF) in many African countries can exert indirect selection pressure on mosquito’s larvae. For example, indoor residual spraying of DDT for malaria control was suspected of favoring the selection of DDT resistance in *Anopheles* [11-13] as well as in *Cx. quinquefasciatus* [14,15]. Contamination of larval breeding sites by insecticides used in agriculture (for example in cotton and vegetables) has also been shown to select for DDT and pyrethroid resistance in malaria vectors [13-16].

In Benin, for the past 10 years, insecticides of the organophosphate (OP) and pyrethroid (PY) groups have been intensively utilized by farmers for pest control and also, in public health as the main strategy to control malaria vectors [11]*.* It is possible that larvae of *Culex* spp which are sympatric with *Anopheles* larvae may be affected by the wide use of these insecticides and developed resistance even though these species were not being targeted.

In Benin, there is more information on insecticide resistance status of malaria vectors compared to Culicines.

Therefore, for an implementation of a vector control program against *Cx. quinquefasciatus,* there is need to have as much information as it is available for these vectors.

The present study aims to assess the resistance status of *Cx. quinquefasciatus* to carbamates, pyrethroids and organochlorine and discuss the implications for vector control in southern and northern Benin.

Data generated from this study will be useful to know if resistance of *Cx. quinquefasciatus* to the insecticides above will jeopardize or not the efficacy of Insecticide Residual Spray (IRS) and Long-Lasting Insecticide-treated Nets (LLINs) on which, most African countries including Benin, rely to reduce malaria transmission.

## Methods

### Methods

#### Study areas

**Figure 1:**
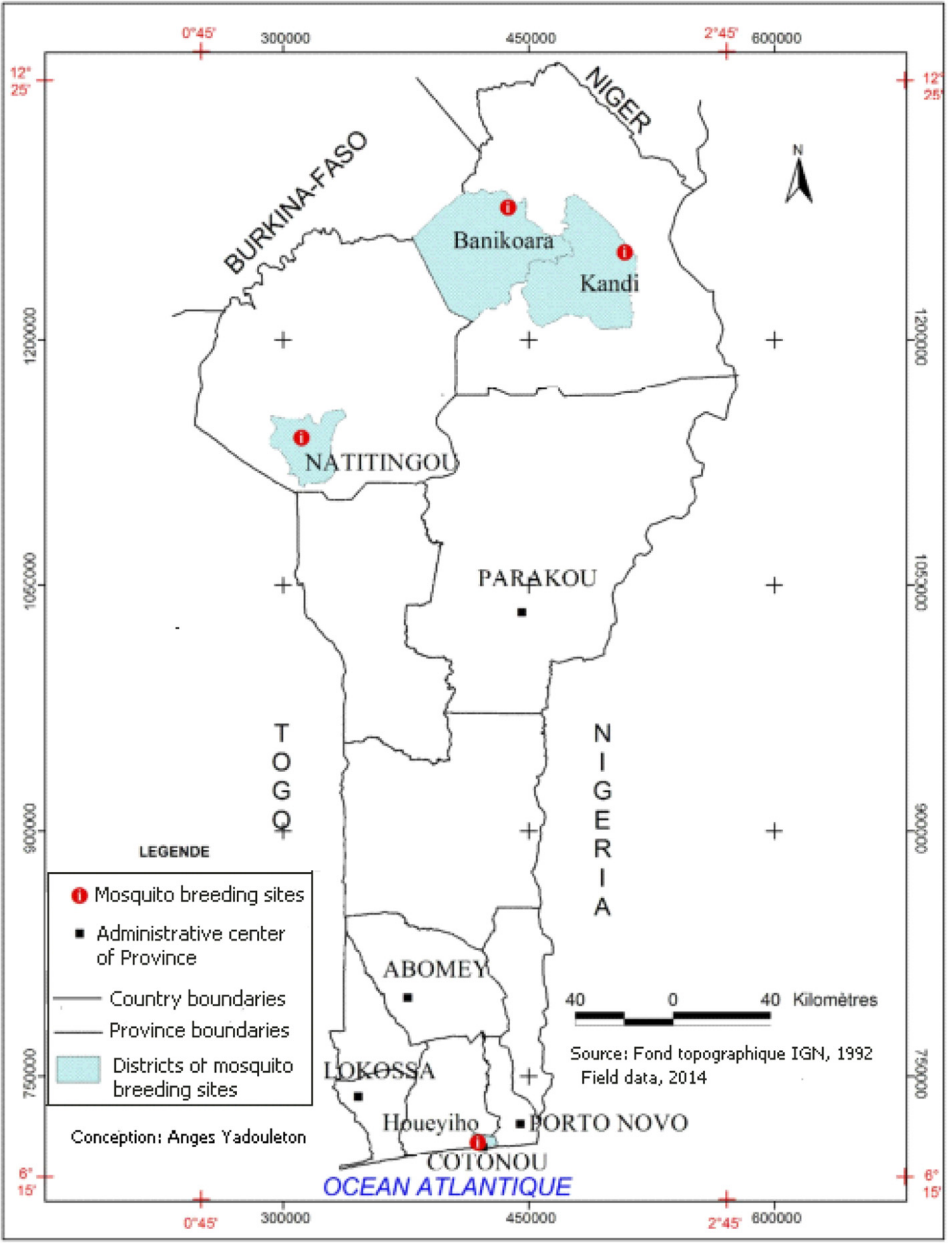
**Map of Benin showing the study sites.**

The study was conducted from January to December 2013 in Benin. Four contrasting localities of the country were selected for mosquito collection on the basis of variation in agricultural production, use of insecticides and/or ecological settings (Figure 1). One rice production area located at Kandi (2°95 E, 11°16 N) with 350 hectares (ha) located in the West-North of Benin; an urban vegetable farming area at Houeyiho (6°45’N and 2°31’E) in southern Benin with 14 ha in size and shared between five local cooperatives of approximately 2,000 farmers; a cotton growing area at Banikoara (2°59 E, 11°31 N) with 50 ha in West-North of Benin and a cereal growing area located at Natitingou (1°23 E, 10°18 N) with 5 ha in East-North of Benin.

In fact, at Banikoara, in the cotton production areas, six pesticide treatments were applied by farmers 45 days after seeding and at two weeks intervals from flowering [17]. At Houeyiho, the increase in vegetable farming in this area has led to the use of insecticides in an improper manner to control vegetable pests [17]. However, at Kandi and Natitingou, no insecticide was used by farmers.

The southern Benin is characterized by a tropical coastal Guinean climate with two rainy seasons (April–July and September–November). The main annual rainfall is more than 1300 mm. The middle part of the country (Parakou) is tropical Sudano-Guinean climate with an average rainfall of 1100 mm per year. The northern part (Kandi, Natitingou and Banikoara) is characterized by a Sudanian climate (semiarid) with only one rainy season per year (main annual rainfall is 900 mm).

Agricultural practices in those farms create numerous trenches that retain rain and water from irrigation systems. These stagnant waters provide suitable breeding sites for mosquitoes, particularly *Cx. quinquefasciatus*.

#### Mosquito collection

*Cx. quinquefasciatus* larvae were sampled from polluted drain across the four contrasting localities selected during the dry season. Contrary to the *Anopheles* larvae which lie parallel to the surface of the water, *Cx. quinquefasciatus* larvae hang at an angle to the surface of the water.

Larvae and pupae were collected using the dipping on breeding sites and then kept in separate labeled bottles for each locality. Larval samples were reared up to adult emergence at the CREC (Centre de Recherche Entomologique de Cotonou, Benin) insectary for further bioassay tests.

### Insecticide susceptibility test

From each site, 150 females mosquitoes aged 2–5 days old were exposed to diagnostic doses of various insecticides for susceptibility tests using insecticide-impregnated papers, as described by the standard WHO testing protocol [18].

Mortality and knockdown resulting from tarsal contact with insecticide-treated filter papers were measured using WHO test kits [18]. The tests were carried out using deltamethrin (0.05%), permethrin (0.75%), DDT (4%) and bendiocarb (0.1%). Four batches of 25 unfed females, aged 2–5 days, were exposed to impregnated papers for 1 h. The number of knock down mosquitoes was recorded every 10 min. Tests with untreated papers that served as control were run in parallel. At the end of the exposure period, mosquitoes were transferred into tubes with untreated white filter papers (known as holding tubes) and allowed a 24 h recovery period. All mosquitoes were provided with 10% glucose water during the 24 h recovery period. Mortality rate was recorded after 24 h. Dead and surviving mosquitoes from this bioassay were separately kept in Carnoy solution at −20°C for further molecular characterization.

### PCR detection of the Knock-down mutation

In each site, 40 survivors of mosquitoes from susceptible tests for each insecticide were used for PCR assays. Each mosquito was extracted using a modified salt-extraction, with total DNA from each mosquito extraction resuspended in 50 μl dH2O (Kent *et al*. [19]). Head + thorax extractions were used to genotype samples for the *kdr* allele, using a Polymerase chain reaction diagnostic test for detection of *kdr* “Leu-phe” mutations following the protocol described by Martinez-Torres *et al.* [20]. PCR conditions are as follows: each 25 μl reaction contained 1X PCR buffer, 100 μM each dNTPs, 75 pmol CxRev primer, 75 pmol forward primer, 2.0 U Taqpolymerase, and 1.5 μl DNA template. Thermocycler conditions consisted of an initial denaturation step of 95°C for 2 minutes; 50 cycles of 94°C for 30 s, 55°C for 1 min, 72°C for 45 s; followed by a final extension of 72°C for 5 min.

Mosquitoes were screened for insensitive acetylcholinesterase (*ace-1*) by the PCR-RFLP method of Weill *et al.* [21]. PCR products were digested overnight for the RFLP, and run on 2% agarose gel.

#### Data interpretation

Mortality rates were corrected using Abbott’s formula when control mortality was above 5% [22]. The resistant status of mosquito samples was determined according to the WHO criteria [18]. Following the WHO protocol, mortality of less than 80%, indicate resistance, while those greater than 98% indicate susceptibility. Mortality between 80%-98%, suggests the possibility of resistance that needs to be verified.

The resistance allele frequency at the *kdr* and *ace-1* locus was calculated using Genepop software (version 3.3) as described by Raymond and Rousset [23].

## Results

### Resistance status

Table 1 shows the insecticide resistance status of *Cx. quinquefasciatus* populations from the four contrasting localities, compared with the susceptible reference strains SLAB.

**Table 1:** **Mortality of the wild populations of** ***Culex. quinquefasciatus*** **from the four study sites after exposure to organochlorine (DDT = 4%), pyrethroids (permethrin = 0.75% and deltamethrine 0.05%) and carbamate (bendiocarb = 0.1%)**

**Population**	**Location**	**Insecticide**	**N**	**% Mortality [Cl95]**	**Resistance status**
**Sites**					
Banikoara	West-North	DDT	100	4 [−3.68-11.68]	R
		Permethrin	100	4 [−3.68-11.68]	R
		Deltamethrin	100	28 [10.40- 45.60]	R
		Bendiocarb	100	52 [32.42- 71.58]	R
Kandi	West-North	DDT	100	12 [− 0.74-24.74]	R
		Permethrin	100	20 [4.32- 35.68]	R
		Deltamethrin	100	24 [24.54- 63.46]	R
		Bendiocarb	100	72 [54.40- 89.60]	R
Natitingou	East-North	DDT	100	12 [− 0.74-24.74]	R
		Permethrin	100	24 [7.26- 40.74]	S
		Deltamethrin	100	48 [28.42- 67.58]	R
		Bendiocarb	100	76 [59.26- 92.74]	R
Houeyiho	South	DDT	100	8 [−2.63 -18.63]	R
		Permethrin	100	8 [−2.63 -18.63]	R
		Deltamethrin	100	32 [13.71- 50.29]	R
		Bendiocarb	100	60 [40.80- 79.20]	R
*Cx*. SLAB		DDT	100	100	S
		Permethrin	100	100	S
		Deltamethrin	100	99	S
		Bendiocarb	100	99	S

*Cx. quinquefasciatus* mosquitoes from all four study areas were resistant to all tested insecticides. Strong resistance was found when *Cx. quinquefasciatus* populations from the four study areas were exposed to pyrethroid and organochlorine. The lowest mortality was observed from the north to the south with the use of DDT where mortality was ranging from 4 to 12%. With the two pyrethroids, the mortality was ranging from 4 to 24% for permethrin and 24 to 48% for deltamethrin. For carbamate, moderate mortality rates was observed after exposing these populations of *Cx. quinquefasciatus* to bendiocarb with mortality rates ranging from 52 to 76%.

In contrast, no alive mosquitoes were recorded when the susceptible strain (SLAB) was exposed to diagnostic doses of various insecticides for susceptibility tests using insecticide-impregnated papers above.

### Detection of resistance genes by PCR

Allele and genotype frequencies at knock-down resistance (*kdr*) and acetylcholinesterase (*ace-* 1) loci of *Cx. quinquefasciatus* from the four study areas are shown in Table 2.

**Table 2:** **Frequency of** ***Kdr*** **and** ***Ace1R*** **mutations in** ***Cx*** **.** ***quinquefasciatus populations*** **from the study sites**

	***Kdr*** **mutation**				***Ace. 1*** **mutation**			
**Locality**	**SS**	**RS**	**RR**	**F(R)**	**SS**	**RS**	**RR**	**F(R)**
Banikoara (102)	4	24	74	0.84	30	08	9	0.27
Houeyiho (92)	3	34	61	0.79	24	06	6	0.25
Kandi (92)	20	40	32	0.56	30	03	3	0.13
Natitingou (92)	24	38	30	0.54	32	04	2	0.10

The highest frequency of *kdr* mutation was recorded from the populations of Banikoara and Houeyiho sites from 0.84 - 0.79 respectively and the lowest 0.56 and 0.54, respectively for Kandi and Natitingou.

An analysis of variance (ANOVA) performed on the frequencies of *kdr* from areas where farmers used insecticides compared to those where no insecticide is use showed that, resistance allele frequency was significantly higher in areas where farmers used insecticides for pest control (Banikoara and Houeyiho) than in those where no insecticide is used (Kandi and N’dali) (p < 0.05.). Moreover, despite the low frequency of *ace.1* found in *Cx. quinquefasciatus* in all sites, an analysis of variance (ANOVA) performed on the frequencies of *ace.1* from areas where farmers used insecticides compared to those where no insecticide is use showed a similar trends with the *kdr* frequencies.

## Discussion

The current study reports the insecticide susceptibility/resistance status of adult *Cx. quinquefasciatus* to organochlorine (DDT), pyrethroids (permethrin, deltamethrin) and carbamate (bendiocarb) in four agro-climatic zone settings in Benin.

Based on the WHO criteria for characterizing insecticide resistance/susceptibility, results from our study sites showed that *Cx. quinquefasciatus* has developed resistance to orgonochlorine, pyrethroids and carbamate. The resistance is very strong with DDT, permethrin and deltamethrin with mortality rates less than 13%, but moderate with bendiocarb with 70% as an average mortality.

In fact, the widespread resistance to DDT and pyrethroid in the four sites can be explained by a long-standing, massive use of DDT house-spraying in several districts of the country during the WHO malaria eradication program in the 1950s [24]. Moreover, the rapid expansion of urban agriculture couple with cotton production in West Africa could be one of the major factors that contribute to a large distribution of pyrethroid resistance in *Cx. quinquefasciatus* [11].

Pyrethroids have been extensively used in agriculture since 1980s [25] particularly in cotton and vegetable fields in Benin. In fact, cotton and vegetable cultivation require intensive use of pesticides including insecticides belonging to the two main classes recommended for vector control in public health (organophosphates and pyrethroids) and which mostly were used indiscriminately to control vegetable and cotton pests. During the treatments, insecticide residues in cotton or vegetable fields are washed into mosquito breeding sites thus exerting a huge selection pressure on mosquito larval populations, which resulted in the emergence of insecticide resistance in *Cx. quinquefasciatus* [11]. This may be the most likely cause of selection on strong resistance in *Cx. quinquefasciatus* to pyrethroids and DDT, particularly in cotton and vegetable growing areas.

Moreover, the massive free campaign of bed nets impregnated with permethrin and deltamethrin as the major control strategy against *Wuchereria bancrofti* transmitted by *Cx. quinquefasciatus* [26,27] could also explain the resistance of *Cx. quinquefasciatus* to pyrethroids*.* This hypothesis has been confirmed by Czeher *et al*. [28] with the increasing of *Leu-Phe knockdown resistance* mutation in *Anopheles gambiae* from Niger following a nationwide long lasting insecticide-treated nets implementation.

This result on insecticide resistance confirmed previous field surveys on *Cx. quinquefasciatus* in Benin conducted by Corbel *et al*. [11].

Therefore, the pyrethroid resistance observed in this part of Benin on *Cx. quinquefasciatus* may seriously jeopardize the efficacy of IRS and LLINs on which most African countries including Benin, rely to reduce malaria transmission.

The presence of *ace-1* mutation particularly in high level in samples from treatments areas could be explained by the intense use of organophosphates by farmers for pests control in agriculture [17] and also in public health for IRS activities [12] in this part of Benin.

With pyrethroid and carbamate resistance spreading in *Cx. quinquefasciatus*, the current findings will help for decision making in the National Malaria Control Program particularly in the choice of insecticide to use during campaigns of Indoor residual spraying in Benin.

The challenge to find effective strategies to manage insecticide resistance in mosquitoes remains a high priority and an urgent need particularly in Benin where pyrethroid resistance has been wildly spread.

Base on the limited numbers of insecticides available for vector control, a rational use of insecticides or mosaic strategy can be adopted to delay development of resistance in mosquitoes in Benin.

Therefore routine surveillance of insecticide susceptibility/resistance in wild populations of *Cx. quinquefasciatus* across different ecological zones in Benin is very urgent for effective resistance management strategy for the efficacy of IRS and LLINs.

## Conclusion

These findings showed that wild populations of *Cx. quinquefasciatus* have developed resistance against pyrethroids, organochlorine and carbamate.

This situation of resistance may seriously jeopardize the efficacy of Insecticide Residual Spray (IRS) and Long-Lasting Insecticide nets (LLINs) on which, most African countries including Benin, rely to reduce malaria transmission.
